# Correlations between First 72 h Hypophosphatemia, Energy Deficit, Length of Ventilation, and Mortality—A Retrospective Cohort Study

**DOI:** 10.3390/nu14071332

**Published:** 2022-03-23

**Authors:** Liran Statlender, Orit Raphaeli, Itai Bendavid, Moran Hellerman, Ilya Kagan, Guy Fishman, Pierre Singer

**Affiliations:** 1 Department of General Intensive Care, Beilinson Hospital, Rabin Medical Center, Petah Tikva 4941492, Israel; itaibd@clalit.org.il (I.B.); moranhe@clalit.org.il (M.H.); ilyak@clalit.org.il (I.K.); guyfi@clalit.org.il (G.F.); psinger@clalit.org.il (P.S.); 2 Industrial Engineering and Management, Ariel University, Ari’el 4077625, Israel; oritre@clalit.org.il; 3 Institute for Nutrition Research, Felsenstein Medical Research Center, Petah Tikva 4941492, Israel

**Keywords:** hypophosphatemia, length of ventilation (LOV), energy deficit

## Abstract

Introduction. Hypophosphatemia may prolong ventilation and induce weaning failure. Some studies have associated hypophosphatemia with increased mortality. Starting or restarting nutrition in a critically ill patient may be associated with refeeding syndrome and hypophosphatemia. The correlation between nutrition, mechanical ventilation, and hypophosphatemia has not yet been fully elucidated. Methods. A retrospective cohort study of 825 admissions during two consecutive years was conducted. Using the electronic medical chart, demographic and clinical data were obtained. Hypophosphatemia was defined as a phosphate level below 2.5 mg/dL (0.81 mmol/L) in the first 72 h of ICU admission. Comparisons between baseline characteristics and outcomes and multivariate analysis were performed. Results. A total of 324 (39.27%) patients had hypophosphatemia during the first 72 h of ICU admission. Patients with hypophosphatemia tended to be younger, with lower APACHE-II, SOFA24, and ΔSOFA scores. They had a longer length of stay and length of ventilation, more prevalent prolonged ventilation, and decreased mortality. Their energy deficit was lower. There was no effect of hypophosphatemia severity on these results. In multivariate analysis, hypophosphatemia was not found to be statistically significant either with respect to mortality or survivor’s length of ventilation, but lower average daily energy deficit and SOFA24 were found to be statistically significant with respect to survivor’s length of ventilation. Conclusion. Hypophosphatemia had no effect on mortality or length of ventilation. Lower average daily energy deficit is associated with a longer survivor’s length of ventilation.

## 1. Introduction

Hypophosphatemia is a common disorder in the critically ill [1,2], found in 15–35% [2,3] of critically ill patients. It has many potential causes, including sepsis [4], refeeding syndrome [5], burns [6], continuous renal replacement therapy (CRRT) [7,8], respiratory alkalosis [9], malnutrition [3], and alcoholism [10].

There is no agreed cutoff value for the phosphate level that defines hypophosphatemia. A meta-analysis by Sin et al. [2] noted several cutoff levels between 1.5–2.9 mg/dL (0.48–0.94 mmol/L). Recent papers have suggested the cutoff of 2.5 mg/dL (0.81 mmol/L) for hypophosphatemia, 2 mg/dL (0.65 mmol/L) for moderate hypophosphatemia, and 1 mg/dL (0.32 mmol/L) for severe hypophosphatemia [3,11]. An earlier cutoff level of 2 mg/dL for hypophosphatemia was suggested as it stands three standard deviations lower than the population mean [12]. The prevalence of hypophosphatemia varies based on the cutoff values used. It also varies between general hospital and ICU populations. A 2005 review [13] found phosphate levels below 1 mg/dL in almost 10% of ICU patients, and 30% of this population had phosphate levels below 2.5 mg/dL, although there was large variability.

In the critically ill patient, hypophosphatemia is associated with several adverse outcomes. While some papers have suggested a correlation between hypophosphatemia and mortality [4,14,15], others have not [1,2,16,17]. Correlation with weaning failure and longer duration of ventilation has been demonstrated [5,18,19], but this is also inconclusive, as some studies have demonstrated no effect or even shorter ventilation duration in patients who had hypophosphatemia [16,20]. The increase in the duration of ventilation seems to prolong hospitalization [2,5,11]. While the prevalence of hypophosphatemia is generally examined over the course of the entire duration of hospitalization, when examining the effects of hypophosphatemia on length of ventilation and mortality, many studies consider only the phosphate level early during the hospitalization [1,5,16].

A correlation between the energy intake of ventilated patients and its effect on phosphate level, length of stay, length of ventilation, and mortality has not been clearly established, and the evidence is conflicting. Some papers describe lower mortality with higher energy intake [21], whereas others have reported the opposite relationship [22]. For example, Arabi et al. found no difference in mortality between standard enteral feeding and permissive underfeeding [23]. Other outcomes, such as length of stay, length of ventilation, and phosphate level changes, may be associated with increased energy delivery to the patient [21,24]. The impact of each of the possible factors (baseline characteristics, prognostic scores at admission, energy administration proportional to patient need, and hypophosphatemia) on these outcomes has not been established. This study aimed to better describe some of these correlations. Since we have observed a correlation between energy delivery and phosphate level, we planned to examine the effect of energy intake and hypophosphatemia on patients’ major outcomes, i.e., mortality and length of ventilation.

## 2. Materials and Methods

This study was approved by the ethics committee of Rabin Medical Center.

We conducted a retrospective analysis of data collected from the electronic medical charts of all ventilated patients who were admitted to a 16-bed general mixed medical-surgical adult ICU over two consecutive years (1 January 2019 through 31 December 2020). For patients who were admitted more than once in the ICU, only the first admission was used.

Data obtained included gender, age at admission, body mass index (BMI), hospitalization dates, length of stay, readmissions, the acute physiology and chronic health evaluation II (APACHE-II) score, the sequential organ failure assessment (SOFA) score at 24 h and 72 h from admission, and its renal component score, changes in the SOFA score (ΔSOFA) at 24 and 72 h from admission, ICU admission category (medical, surgical, trauma, obstetrics, transplantation), daily minimal phosphate level during the first 72 h of admission, blood gas results during the first 72 h of admission (pH, pCO2, bicarbonate level, and base excess), length of ventilation, total energy provided during hospitalization, daily average target energy estimated by the Faisy-Fagon equation [25,26], and ICU outcome (discharge to a hospital ward, discharge to a rehabilitation center, or death; patients who were discharged alive from ICU but died in subsequent ICU readmission (within the same hospital admission) were considered as mortality in the first ICU admission).

For each patient, we calculated the time-weighted average of pH, pCO_2_, bicarbonate, base excess, and kidney function SOFA score during the first 72 h from admission. We used the kidney SOFA score as a surrogate to kidney function, as creatinine levels do not reflect urine volume changes, and are affected by muscle tissue waste, and dialysis status.

For each ventilated patient we calculated the energy deficit by subtracting the total amount of energy administered from the average resting energy expenditure calculated by Faisy-Fagon’s formula [26] multiplied by the length of stay (days). This deficit was adjusted to the length of stay to define the average daily energy deficit (AvgDED).

Prolonged ventilation was defined as ventilation that lasted more than a week, based on weaning difficulty classification [27].

Mortality occurrence within the first week of ICU admission shortens ventilation duration. To avoid this masking, we performed two analyses: (1) a composite outcome, Vent7Mortality, which includes all those patients who died and those who survived but were ventilated for more than 7 days; (2) comparison of length of ventilation only of the survivors.

Statistical analysis was performed using SAS vs. 9.4 (SAS Institute, Cary, NC, USA). We compared baseline characteristics and patient outcomes between patients who were found to have hypophosphatemia (phosphate lower than 2.5 mg/dL) in the first 72 h of ICU admission and those who did not (in case of several results of low phosphate during that period, the lowest level was used). Comparisons were made using the student’s *t*-test or Mann–Whitney for numerical variables and the chi-square test for categoric variables. A multivariate logistic regression was performed to define the odds ratio (OR) for prolonged ventilation, mortality, and Vent7mortality.

## 3. Results

### 3.1. Baseline Characteristics

**Figure 1:**
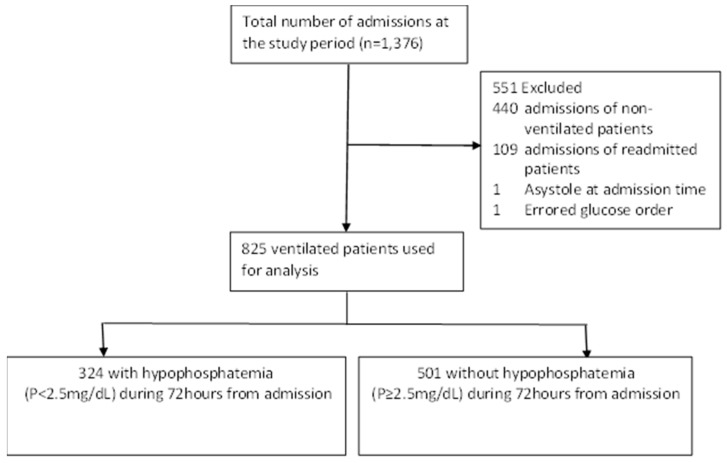
Flow chart of study design.

During the study period, there were 1376 admissions to our ICU. Excluding 440 admissions of non-ventilated patients, 109 readmissions, and 2 other admissions (one with asystole on admission who did not survive, and one who had an error in a glucose admission order that altered the number of calories prescribed)—825 patients were used for the analysis (Figure 1).

Three hundred twenty-four (39.27%) patients had hypophosphatemia during the first 72 h of ICU admission (that is, a phosphate level lower than 2.5 mg/dL during this period). Of these patients, one hundred seventy-four (21.10%) had phosphate levels less than 2 mg/dL, and twenty-one (2.55%) had phosphate levels less than 1 mg/dL.

Table 1 shows the baseline characteristics of the patients, grouped by hypophosphatemia level. Patients with hypophosphatemia were younger (55.76 vs. 60.03, *p* < 0.01), had lower APACHE-II score (20.35 vs. 23.38, *p* < 0.001), lower SOFA24 score (7.53 vs. 8.91, *p* < 0.001), and lower ΔSOFA (−1.08 vs. −0.40, *p* < 0.001). Their Kidney SOFA score was 1 point lower than the non-hypophosphatemia group; and they tended to be slightly more alkalemic (pH 7.4 vs. 7.36, *p* < 0.001), mainly due to a slightly higher bicarbonate level (26.1 mmol/L vs. 23.5 mmol/L, *p* < 0.001). There was also a difference in the admission reason to ICU between patients with hypophosphatemia and those without (*p* < 0.001)—primarily a greater number of trauma admissions in the hypophosphatemia group. There was no significant difference between patients with and without hypophosphatemia regarding sex and BMI.

### 3.2. Admission Outcomes

Table 2 shows patient admission outcomes.

The length of ICU stay was significantly higher in the hypophosphatemia group compared with the non-hypophosphatemia group (10.19 days vs. 8.11 days, *p* < 0.001).

The length of ventilation was longer in the hypophosphatemia group compared with the non-hypophosphatemia group (8.90 days vs. 6.69 days, *p* < 0.001). Prolonged ventilation was more frequent among patients with hypophosphatemia than among those without (43.83% vs. 29.74%, *p* < 0.01).

The average daily energy deficit was less negative (i.e., a smaller energy deficit) in the hypophosphatemia group compared with the non-hypophosphatemia group (−959 Kcal/day vs. −1175 Kcal/day, *p* < 0.01).

The mortality rate was lower in the hypophosphatemia group (24.07% vs. 43.71%, *p* < 0.01). The unadjusted OR for mortality in the hypophosphatemia group was 0.41 (95% CI 0.3–0.56).

The composite outcome Vent7mortality was not significantly different between the hypophosphatemia and non-hypophosphatemia groups (55.25% vs. 59.68% respectively, *p* = 0.21). The unadjusted OR for Vent7mortality was 0.83 (95% CI 0.63–1.11).

Concerning hypophosphatemia severity, the only difference in any of baseline characteristics between patients with mild, moderate, and severe hyperphosphatemia was SOFA24, which was lower in patients with mild hypophosphatemia than in those with moderate and severe hypophosphatemia (6.92, 7.98, and 8.56 respectively; *p* < 0.03 for mild vs. moderate, and *p* < 0.04 for mild vs. severe). There was no difference in outcomes (length of stay, length of ventilation, prolong ventilation, AvgDED, mortality, and Vent7Mortality) according to hypophosphatemia severity. See Supplementary Table S1.

### 3.3. Survivors’ Analysis

Among the 528 survivors, there was no difference between the hypophosphatemia group and non-hypophosphatemia group regarding age, sex, BMI, or APACHE-II (Table 3).

SOFA24 was lower in the hypophosphatemia group (7.06 vs. 8.11, *p* = 0.001); kidney SOFA score was almost 1 point lower in the hypophosphatemia group; pH was slightly higher in the hypophosphatemia group (7.41 vs. 7.39, *p* < 0.001) mainly due to slightly higher bicarbonate level (26.3 mmol/L vs. 24.8 mmol/L, *p* < 0.001); AvgDED was higher (−983.6 vs. −1177.76, *p* < 0.001). There was also a significant difference in the admission category (more trauma patients in the hypophosphatemia group).

The length of ventilation was higher in the hypophosphatemia group (8.53 days vs. 6.17 days, *p* = 0.002). Prolonged ventilation was more frequent among surviving patients with hypophosphatemia than among surviving patients without hypophosphatemia (41.07% vs. 28.37%, respectively, *p* = 0.02). Unadjusted OR for prolonged ventilation in the hypophosphatemia group was 1.75 (95% CI 1.22–2.52).

### 3.4. Multivariate Analysis

In multivariate analysis for mortality (Table 4a), hypophosphatemia was not found as a significant covariate (OR 0.77, 95% CI 0.42–1.44). Among covariates that were examined, age, SOFA24, ΔSOFA, AvgDED, and trauma admission category were found to be significant. These results were not changed after incorporating into the model acid-base data.

Hypophosphatemia was not found to be a significant covariate regarding the survivor’s ventilation length (Table 4b). Among covariates that were examined, female sex, SOFA24, ΔSOFA, AvgDED, and obstetrics category of admission were found significant. These results were not changed after incorporating into the model acid-base data.

Admission to ICU due to trauma was significantly more prevalent in the hypophosphatemia group. As the mortality rate of trauma patients with hypophosphatemia was lower, an analysis of only trauma patients was performed. Similar to the whole cohort, in the trauma patient population, patients with hypophosphatemia experienced longer ventilation periods (OR 2.45, 95% CI 1.11–5.41). A trend towards lower mortality of trauma patients with hypophosphatemia was found (OR 0.43, 95% CI 0.18–1.04), and hypophosphatemia was not found to be a significant covariate to the composite outcome of Vent7Mortality in trauma patients (OR 1.55, 95% CI 0.77–3.09). Multivariate analysis found neither baseline characteristics nor hypophosphatemia to be significant covariates for mortality or Vent7mortality, but AvgDED was found to be a significant covariate to Vent7Mortality (See Supplementary Table S2).

## 4. Discussion

### 4.1. Baseline Charectaristics & Energy Delivery

Hypophosphatemia is generally associated with poor outcome measures such as mortality rate, prolonged ventilation periods, and prolonged ICU and hospital stays [2,4]. Based on the commonly cited cutoff value of 2.5 mg/dL [3,11], we compared two groups of patients—those with hypophosphatemia (at least one result lower than 2.5 mg/dL during the first 72 h of ICU admission) and those without. The baseline characteristics were different between these groups—patients with hypophosphatemia were younger, had lower APACHE-II, SOFA24, and ΔSOFA scores. They had lower kidney SOFA score and tended to have slightly higher pH mainly due to slightly elevated bicarbonate level (both parameters during the first 72 h of admission). A higher proportion of trauma admission category was noted among patients with hypophosphatemia.

As for the acid-base difference, although statistically significant, its clinical importance is likely negligible, as the absolute difference is minimal (0.04 difference at the pH level, and 2.5 mmol/L difference in the bicarbonate level).

The higher kidney SOFA score of the patients without hypophosphatemia is probably explained by hyperphosphatemia of kidney injury. Hyperphosphatemia is correlated with other severity scores such as APACHE-II and SOFA [28].

There is no clear explanation for the difference in these baseline characteristics between patients with hypophosphatemia and those without. Some of these differences (younger age [16] and lower prognostic score [20]) have been described in patients with hypophosphatemia, however other studies have not found such differences [1,5,14].

Patients with hypophosphatemia also received more energy during their hospitalization (as evident by the lower AvgDED). It is possible that nutritional support was started earlier in the younger and less severe patients (as evident by the less negative AvgDED), and therefore more of these patients developed refeeding syndrome and early hypophosphatemia.

Another possible explanation is that hypophosphatemia may be a marker of recovery. In fulminant hepatic failure, and during recovery after hepatectomy, hypophosphatemia is a well-established marker of a favorable prognosis [29,30]. At the cellular level, hypophosphatemia is correlated with higher levels of intracellular ATP. This has been described specifically in liver diseases, and not in general critically ill patients, and therefore the generalizability of this hypothesis has not been previously substantiated. If this hypothesis is correct, we can further posit that hypophosphatemia may be associated with a more favorable prognosis (similar to a lower APACHE-II and SOFA). This may explain the higher occurrence of trauma patients in the hypophosphatemia group.

### 4.2. Mortality & Prolonged Ventilation

Although hypophosphatemia was found to be significant as a covariate for lower mortality rate in univariate analysis, this could not be established in multivariate analysis. The absence of hypophosphatemia effect on mortality is concurrent with some of recent literature [1,2,16,17], but this issue is controversial, as other papers have demonstrated increased mortality [4,14,15] in patients with hypophosphatemia. The lower mortality rate in the hypophosphatemia group may be explained by the younger age and lower severity scores of these patients. AvgDED was found to be significant in the multivariate analysis for mortality (OR CI 0.999–1.0). This might suggest a beneficial effect of energy delivery to the patient.

With respect to the composite outcome combining mortality and prolonged ventilation, there was no difference demonstrated between the groups. Bearing in mind the lower mortality rate in the hypophosphatemia group, the lack of difference between the groups with respect to this combined composite outcome may be attributable to a higher prevalence of prolonged ventilation, both in the entire cohort, and in the survivors alone. Hypophosphatemia has been demonstrated to be correlated with prolonged ventilation and extubation failure [5,18,19]. As we did not collect daily fluid status, number of extubation failures or failed spontaneous breathing trails, we cannot better characterize the relationship between prolonged ventilation and hypophosphatemia in our cohort. Whether the similarity in Vent7Mortatilty between groups (i.e., lower mortality and higher prolonged ventilation rate in the hypophosphatemia group, and the opposite outcomes in the non-hypophosphatemia group) is a random finding seen in this cohort or not will require further evaluation.

### 4.3. Survivor’s Length of Ventilation

While the length of ventilation in the hypophosphatemia group was longer in univariate analysis, this was not demonstrated in multivariate analysis. Differences observed between groups were in the SOFA24 score, which was lower in the hypophosphatemia group; the admission category, with a higher proportion of trauma patients in the hypophosphatemia group; and AvgDED, which was less negative in the hypophosphatemia group.

In multivariate analysis, significant covariates were SOFA24, ΔSOFA, AvgDED, female sex, and an admission category of obstetrics.

In the hypophosphatemia group, ventilation duration was longer (almost 2.5 days), whereas SOFA24 was lower (1.06 difference), as compared to the non-hypophosphatemia group. However, SOFA24 was found to be a significant covariate with respect to prolonged ventilation. Longer ventilation (and hospitalization) would be expected in more severe diseases, implied by a higher SOFA24, especially among surviving patients. As such, the difference of 1.06 points in the SOFA score between the groups is likely clinically insignificant. Moreover, longer ventilation is more common among severe trauma patients, who need longer ICU hospitalization due to the nature of their injuries—first until stabilization, and later until achieving definite surgical solution and regaining full consciousness.

The covariate significance of obstetric admissions must be interpreted with caution as the number of obstetric admissions was very low in our cohort, representing only 10 of 825 admissions. Moreover, these patients are generally young, previously healthy women who were ventilated for shorter periods, most often following emergency caesarian section due to severe preeclampsia. The statistical significance must be interpreted in the context of the small number of patients and the specific characteristics in this group.

The other covariate that may explain the length of ventilation among survivors in the multivariate analysis is the average daily caloric deficit (OR CI 1.003–1.006). This finding might be explained by the appearance of the refeeding syndrome [11], as more energy is delivered to the patient. Due to refeeding syndrome, patients might suffer from weakness, and not be successfully weaned. Our ICU is very mindful of refeeding syndrome—both in terms to prevention and treatment, as evident from the very low incidence of severe hypophosphatemia in our cohort (2.55%). It is possible that aggressively screening for hypophosphatemia and correcting it, especially several hours after initiating or re-initiating nutrition (as done regularly in our ICU), mitigated the prevalence of severe hypophosphatemia. This higher energy delivery might have caused the pathological process leading to the refeeding syndrome, in turn translating into longer ventilation periods. However, as the hypophosphatemia was corrected aggressively, it was not found to be a significant covariate to the length of ventilation.

### 4.4. The Interaction of Energy Delivery, Hypophsphatemia, & Patient Outcomes

The relationship between hypophosphatemia and energy delivery has been stressed by Doig et al. A protocolized energy restriction to ICU patients suffering from hypophosphatemia resulted in significant improvements in overall survival time and mortality at 60 days follow-up [31]. These findings show that in terms of correction of hypophosphatemia, it is recommended to decrease the energy administration simultaneously, as advised by ESPEN, the European Society of Clinical Nutrition and Metabolism [32]. Nevertheless, our analysis suggests that energy administration with hypophosphatemia does not cause increased mortality and may be safe in a similar patient cohort. The correlation between mortality and phosphate level is generally considered as U shaped: higher phosphate levels are generally associated with kidney injury (as was noticed in our cohort), which by itself is a factor for increased mortality; lower phosphate levels may be related to energy intake [33].

The increased length of ventilation was explained by higher energy administration in our cohort. Overfeeding is related to increased length of ventilation [24], and so it is not surprising that higher energy delivery, as opposed to hypophosphatemia, is related to longer ventilation duration. It is possible that this effect of longer ventilation duration without increased mortality depends on refeeding syndrome mechanism.

Of note, we did not evaluate in this study whether there is a difference between enteral and parenteral support. As energy delivery to the patient is increased until achieving energy target (whether enteral or parenteral), it seems logical that nutritional support administration would have similar results irrespective of delivery form, but this should be separately assessed.

### 4.5. Limitations

Our work has several limitations. First, it is a retrospective cohort study. Second, we could not use indirect calorimetry to assess resting energy expenditure (REE) and the caloric deficit. Indirect calorimetry is known to better assess REE, while formulas aiming to calculate REE are often inaccurate. However, indirect calorimetry was performed only in minority of the patients (189 measurements in 73 patients (8.85% of the cohort), and therefore we could not use the measurements. Faisy-Fagon formula, used in this study, has been shown to be more accurate than other formulas [25,26,34]. Third, the average daily caloric deficit is only an average calculated over the entire hospitalization, not a clinical parameter. Most energy delivery deficit likely develops at the beginning of the admission, as the patient does not prescribed energy due to hemodynamic instability or severe hypoxemia. Although AvgDED was designed to adjust for the length of stay, in longer hospitalization courses the effect of energy deficits during the first days of the hospitalization is smaller. However, as this factor was found to be significant in most of the analysis, we suggest it should not be ignored.

## 5. Conclusions

Hypophosphatemia had no effect on mortality or the length of ventilation in our cohort of critically ill patients. Lower energy deficits, which are markers of higher energy delivery to the patient, were associated with longer length of ventilation periods among survivors. Further research is needed to better describe this finding.

## Figures and Tables

**Table 1:** Baseline characteristics.

	Total	Without Hypophosphatemia Minimal *p* ≥ 2.5 mg/dL	With Hypophosphatemia Minimal *p* < 2.5 mg/dL	
	825	501 (60.73%)	324 (39.27%)	
Age (years)	58.36 ± 17.58	60.03 ± 16.95	55.76 ± 18.24	*p* < 0.01
Male sex (n, %)	523 (63.39%)	320 (61.19%)	203 (38.81%)	*p* = 0.767
BMI (kg/m^2^)	28.00 ± 6.53	28.08 ± 6.52	27.87 ± 6.55	*p* = 0.654
APACHE II	22.09 ± 7.68	23.38 ± 8.02	20.35 ± 6.84	*p* < 0.001
SOFA24	8.34 ± 3.51	8.91 ± 3.59	7.53 ± 3.23	*p* < 0.001
ΔSOFA24-72	−0.70 ± 2.79	−0.40 ± 2.98	−1.08 ± 2.49	*p* = 0.005
pH	7.38 ± 0.07	7.36 ± 0.8	7.4 ± 0.05	*p* < 0.001
pCO_2_ (mmHg)	42.90 ± 8.05	42.46 ± 8.16	43.57 ± 7.86	*p* = 0.013
HCO_3_^−^ (mmol/L)	24.53 ± 4.37	23.51 ± 4.45	26.11 ± 3.71	*p* < 0.001
Base Excess (mmol/L)	−0.02 ± 5.11	−1.23 ± 5.26	1.83 ± 4.32	*p* < 0.001
Kidney SOFA score	1.05 ± 1.32	1.5 ± 1.39	0.45 ± 0.92	*p* < 0.001
Admission reason (n,%)				
Medical	437 (52.97%)	259 (51.70%)	178 (54.94%)	*p* < 0.001
Surgical	183 (22.18%)	126 (25.15%)	57 (17.59%)	
Trauma	135 (16.36%)	58 (11.58%)	77 (23.77%)	
Obstetrics	10 (1.21%)	9 (1.80%)	1 (0.31)	
Transplantation	60 (7.27%)	49 (9.78%)	11 (3.40%)	

*p* value is based on *t*-test or Mann–Whitney (as appropriate) for numerical variables and chi-square test for categorial variables.

**Table 2:** Outcomes of admissions.

	Total	Without Hypophosphatemia Minimal *p* ≥ 2.5 mg/dL	With Hypophosphatemia Minimal *p* < 2.5 mg/dL	
N	825	501	324	
Length of stay (days)	8.93 ± 9.83	8.11 ± 9.97	10.19 ± 9.48	*p* < 0.001
Length of ventilation (days)	7.55 ± 8.85	6.69 ± 8.65	8.90 ± 8.99	*p* < 0.001
Prolonged ventilation (n, %)	291 (35.27%)	149 (29.74%)	142 (43.83%)	*p* < 0.01
Average daily energy deficit (Kcal/day)	−1091.05 ± 574.41	−1175.88 ± 566.51	−959.89 ± 562.59	*p* < 0.01
Death (n, %)	297 (36.0%)	219 (43.71%)	78 (24.07%)	*p* < 0.01
Vent7Mort (n, %)	478 (57.94%)	299 (59.68%)	179 (55.25%)	*p* = 0.2

*p* value is based on *t*-test or Mann-Whitney (as appropriate) for numerical variables and chi-square test for categorial variables.

**Table 3:** Baseline characteristics and outcomes of survivors only.

	Total	Without Hypophosphatemia Minimal *p* ≥ 2.5 mg/dL	With Hypophosphatemia Minimal *p* < 2.5 mg/dL	
N	528	282	246	
Age (years)	54.60 ± 17.99	55.38 ± 17.48	53.70 ± 18.55	*p* = 0.28
Male sex (n, %)	333 (63.07%)	175 (62.06%)	158 (64.23%)	*p* = 0.61
BMI (kg/m^2^)	28.13 ± 6.59	28.15 ± 6.59	28.11 ± 6.60	*p* = 0.95
APACHE II	19.53 ± 6.81	20.08 ± 7.11	18.99 ± 6.49	*p* = 0.16
SOFA24	7.61 ± 3.22	8.12 ± 3.51	7.06 ± 2.79	*p* = 0.001
ΔSOFA24-72	−1.20 ± 2.87	−1.13 ± 3.11	−1.26 ± 2.64	*p* = 0.74
pH	7.40 ± 0.05	7.39 ± 0.06	7.41 ± 0.04	*p* < 0.001
pCO_2_ (mmHg)	42.8 ± 7.25	42.37 ± 6.72	43.30 ± 7.80	*p* = 0.14
HCO_3_^−^ (mmol/L)	25.5 ± 3.68	24.79 ± 3.69	26.31 ± 3.52	*p* < 0.001
Base Excess (mmol/L)	1.14 ± 4.17	0.31 ± 4.11	2.09 ± 4.05	*p* < 0.001
Kidney SOFA score	0.83 ± 1.26	1.27 ± 1.39	0.38 ± 0.82	*p* < 0.001
Admission reason				
Medical	258 (48.86%)	133 (47.16%)	125 (50.81%)	*p* < 0.001
Surgical	100 (18.94%)	58 (20.57%)	42 (17.07%)	
Trauma	110 (20.83%)	43 (15.25%)	67 (27.24%)	
Obstetrics	10 (1.89%)	9 (3.19%)	1 (0.41%)	
Transplantation	50 (9.47%)	39 (13.83%)	11 (4.47%)	
Average daily energy deficit (Kcal/day)	−1087.30 ± 593.10	−1177.76 ± 594.84	−983.60 ± 547.98	*p* < 0.001
Length of stay (days)	8.87 ± 9.35	7.83 + 9.02	10.07 ± 9.59	*p* < 0.01
Length of ventilation (days)	7.27 ± 8.71	6.17 ± 8.24	8.53 ± 9.08	*p* = 0.002
Prolonged ventilation	181 (34.28%)	80 (28.37%)	101 (41.06%)	*p* = 0.02

*p* value is based on *t*-test or Mann-Whitney (as appropriate) for numerical variables and chi-square test for categorial variables.

**Table 4:** Multivariate analysis. (a) Multivariate analysis for mortality; (b) Multivariate analysis for survivor’s length of ventilation.

(a)				
**Effect**	**OR**	**95% Confidence Limits**		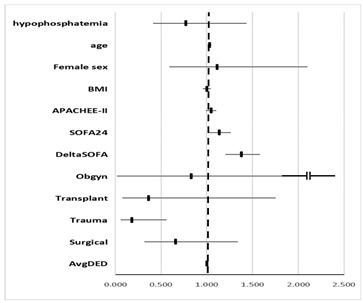
hypophosphatemia	0.773	0.416	1.435	
**age**	**1.028**	**1.007**	**1.049**	
female sex	1.113	0.590	2.099	
BMI	1.000	0.956	1.047	
APACHEE-II	1.046	0.994	1.101	
**SOFA24**	**1.135**	**1.021**	**1.262**	
**ΔSOFA**	**1.379**	**1.203**	**1.580**	
Obgyn (vs. medical)	0.828	0.021	32.872	
Transplant (vs. medical)	0.365	0.076	1.750	
**Trauma (vs. medical)**	**0.183**	**0.060**	**0.562**	
Surgical (vs. medical)	0.656	0.321	1.341	
Average daily energy deficit	1.000	0.999	1.000	
(**b**)				
**Effect**	**OR**	**95% Confidence Limits**		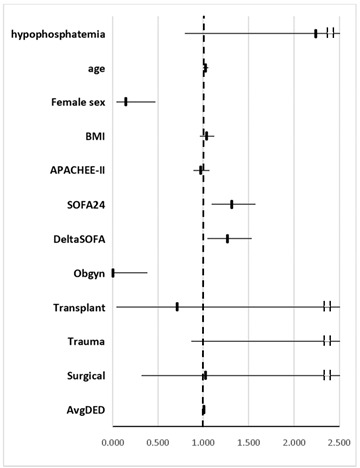
hypophosphatemia	2.242	0.797	6.289	
age	1.026	0.996	1.058	
**female sex**	**0.143**	**0.043**	**0.473**	
BMI	1.039	0.961	1.123	
APACHEE-II	0.973	0.889	1.066	
**SOFA24**	**1.311**	**1.090**	**1.576**	
**ΔSOFA**	**1.267**	**1.045**	**1.537**	
**Obgyn (vs. medical)**	**0.003**	**0.001**	**0.384**	
Transplant (vs. medical)	0.711	0.045	11.339	
Trauma (vs. medical)	3.825	0.866	16.897	
Surgical (vs. medical)	1.025	0.319	3.280	
**Average daily energy deficit**	**1.005**	**1.003**	**1.006**	

Bold covariates are statistically significant.

## Data Availability

The data presented in this study are available on request from the corresponding author. The data are not publicly available due to patients privacy reasons.

## Supplementary Materials

The following supporting information can be downloaded at: https://www.mdpi.com/article/10.3390/nu14071332/s1, Table S1: Baseline characteristics and outcomes according to hypophosphatemia severity; Table S2: Multivariate analysis for trauma patients only.

## References

[1] Suzuki S., Egi M., Schneider A.G., Bellomo R., Hart G.K., Hegarty C. (2013). Hypophosphatemia in Critically Ill Patients. J. Crit. Care.

[2] Sin J.C.K., King L., Ballard E., Llewellyn S., Laupland K.B., Tabah A. (2020). Hypophosphatemia and Outcomes in ICU: A Systematic Review and Meta-Analysis. J. Intensive Care Med..

[3] Berger M.M., Appelberg O., Reintam-Blaser A., Ichai C., Joannes-Boyau O., Casaer M., Schaller S.J., Gunst J., Starkopf J. (2020). ESICM-MEN section Prevalence of Hypophosphatemia in the ICU—Results of an International One-Day Point Prevalence Survey. Clin. Nutr..

[4] Shor R., Halabe A., Rishver S., Tilis Y., Matas Z., Fux A., Boaz M., Weinstein J. (2006). Severe Hypophosphatemia in Sepsis as a Mortality Predictor. Ann. Clin. Lab. Sci..

[5] Marik P.E., Bedigian M.K. (1996). Refeeding Hypophosphatemia in Critically Ill Patients in an Intensive Care Unit. A Prospective Study. Arch. Surg..

[6] Rimaz S., Moghadam A.D., Mobayen M., Nasab M.M., Rimaz S., Aghebati R., Jafaryparvar Z., Rad E.H. (2019). Changes in Serum Phosphorus Level in Patients with Severe Burns: A Prospective Study. Burns.

[7] Pistolesi V., Zeppilli L., Fiaccadori E., Regolisti G., Tritapepe L., Morabito S. (2019). Hypophosphatemia in Critically Ill Patients with Acute Kidney Injury on Renal Replacement Therapies. J. Nephrol..

[8] Hendrix R.J., Hastings M.C., Samarin M., Hudson J.Q. (2020). Predictors of Hypophosphatemia and Outcomes during Continuous Renal Replacement Therapy. Blood Purif..

[9] Paleologos M., Stone E., Braude S. (2000). Persistent, Progressive Hypophosphataemia after Voluntary Hyperventilation. Clin. Sci..

[10] Larsson L., Rebel K., Sörbo B. (1983). Severe Hypophosphatemia—A Hospital Survey. Acta Med. Scand..

[11] Reintam Blaser A., Gunst J., Ichai C., Casaer M.P., Benstoem C., Besch G., Dauger S., Fruhwald S.M., Hiesmayr M., Joannes-Boyau O. (2020). Hypophosphatemia in Critically Ill Adults and Children—A Systematic Review. Clin. Nutr..

[12] Betro M.G., Pain R.W. (1972). Hypophosphataemia and Hyperphosphataemia in a Hospital Population. Br. Med. J..

[13] Gaasbeek A., Meinders A.E. (2005). Hypophosphatemia: An Update on Its Etiology and Treatment. Am. J. Med..

[14] Wang L., Xiao C., Chen L., Zhang X., Kou Q. (2019). Impact of Hypophosphatemia on Outcome of Patients in Intensive Care Unit: A Retrospective Cohort Study. BMC Anesthesiol..

[15] Hoffmann M., Zemlin A.E., Meyer W.P., Erasmus R.T. (2008). Hypophosphataemia at a Large Academic Hospital in South Africa. J. Clin. Pathol..

[16] Federspiel C.K., Itenov T.S., Thormar K., Liu K.D., Bestle M.H. (2018). Hypophosphatemia and Duration of Respiratory Failure and Mortality in Critically Ill Patients. Acta Anaesthesiol. Scand..

[17] Cohen J., Kogan A., Sahar G., Lev S., Vidne B., Singer P. (2004). Hypophosphatemia Following Open Heart Surgery: Incidence and Consequences. Eur. J. Cardio-Thorac. Surg..

[18] Geerse D.A., Bindels A.J., Kuiper M.A., Roos A.N., Spronk P.E., Schultz M.J. (2010). Treatment of Hypophosphatemia in the Intensive Care Unit: A Review. Crit. Care.

[19] Alsumrain M.H., Jawad S.A., Imran N.B., Riar S., DeBari V.A., Adelman M. (2010). Association of Hypophosphatemia with Failure-to-Wean from Mechanical Ventilation. Ann. Clin. Lab. Sci..

[20] Miller C.J., Doepker B.A., Springer A.N., Exline M.C., Phillips G., Murphy C.V. (2020). Impact of Serum Phosphate in Mechanically Ventilated Patients with Severe Sepsis and Septic Shock. J. Intensive Care Med..

[21] Singer P., Anbar R., Cohen J., Shapiro H., Shalita-Chesner M., Lev S., Grozovski E., Theilla M., Frishman S., Madar Z. (2011). The Tight Calorie Control Study (TICACOS): A Prospective, Randomized, Controlled Pilot Study of Nutritional Support in Critically Ill Patients. Intensive Care Med..

[22] Braunschweig C.L., Freels S., Sheean P.M., Peterson S.J., Perez S.G., McKeever L., Lateef O., Gurka D., Fantuzzi G. (2017). Role of Timing and Dose of Energy Received in Patients with Acute Lung Injury on Mortality in the Intensive Nutrition in Acute Lung Injury Trial (INTACT): A Post Hoc Analysis. Am. J. Clin. Nutr..

[23] Arabi Y.M., Aldawood A.S., Haddad S.H., Al-Dorzi H.M., Tamim H.M., Jones G., Mehta S., McIntyre L., Solaiman O., Sakkijha M.H. (2015). Permissive Underfeeding or Standard Enteral Feeding in Critically Ill Adults. N. Engl. J. Med..

[24] Zusman O., Theilla M., Cohen J., Kagan I., Bendavid I., Singer P. (2016). Resting Energy Expenditure, Calorie and Protein Consumption in Critically Ill Patients: A Retrospective Cohort Study. Crit. Care.

[25] Faisy C., Guerot E., Diehl J.-L., Labrousse J., Fagon J.-Y. (2003). Assessment of Resting Energy Expenditure in Mechanically Ventilated Patients. Am. J. Clin. Nutr..

[26] Savard J.-F., Faisy C., Lerolle N., Guerot E., Diehl J.-L., Fagon J.-Y. (2008). Validation of a Predictive Method for an Accurate Assessment of Resting Energy Expenditure in Medical Mechanically Ventilated Patients. Crit. Care Med..

[27] Boles J.-M., Bion J., Connors A., Herridge M., Marsh B., Melot C., Pearl R., Silverman H., Stanchina M., Vieillard-Baron A. (2007). Weaning from Mechanical Ventilation. Eur. Respir. J..

[28] Jung S.-Y., Kwon J., Park S., Jhee J.H., Yun H.-R., Kim H., Kee Y.K., Yoon C.-Y., Chang T.-I., Kang E.W. (2018). Phosphate Is a Potential Biomarker of Disease Severity and Predicts Adverse Outcomes in Acute Kidney Injury Patients Undergoing Continuous Renal Replacement Therapy. PLoS ONE.

[29] Chung P. (2003). Serum Phosphorus Levels Predict Clinical Outcome in Fulminant Hepatic Failure. Liver Transplant..

[30] Pomposelli J. (2001). Life-Threatening Hypophosphatemia after Right Hepatic Lobectomy for Live Donor Adult Liver Transplantation. Liver Transplant..

[31] Doig G.S., Simpson F., Heighes P.T., Bellomo R., Chesher D., Caterson I.D., Reade M.C., Harrigan P.W.J. (2015). Restricted versus Continued Standard Caloric Intake during the Management of Refeeding Syndrome in Critically Ill Adults: A Randomised, Parallel-Group, Multicentre, Single-Blind Controlled Trial. Lancet Respir. Med..

[32] Singer P., Blaser A.R., Berger M.M., Alhazzani W., Calder P.C., Casaer M.P., Hiesmayr M., Mayer K., Montejo J.C., Pichard C. (2019). ESPEN Guideline on Clinical Nutrition in the Intensive Care Unit. Clin. Nutr..

[33] Berger M.M., Reintam-Blaser A., Calder P.C., Casaer M., Hiesmayr M.J., Mayer K., Montejo J.C., Pichard C., Preiser J.-C., van Zanten A.R.H. (2019). Monitoring Nutrition in the ICU. Clin. Nutr..

[34] De Waele E., Opsomer T., Mattens S., Diltoer M., Honoré P.M., Spapen H., Huyghens L. (2013). Pp192-Sun Measured Versus Calculated Resting Energy Expenditure in Critically Ill Adult Patients. Do Mathematics Match the Golden Standard?. Clin. Nutr..

