# Supplementary material for: Correlations between First 72 h Hypophosphatemia, Energy Deficit, Length of Ventilation, and Mortality—A Retrospective Cohort Study
Source: Nutrients. 2022 Mar 23;14(7):1332. doi: 10.3390/nu14071332 (PMC9002762; doi:10.3390/nu14071332)
Supplement: Supplementary file 1 [file nutrients-14-01332-s001.zip › nutrients-1591746-supplementary.pdf]

Table S1. Baseline characteristics and outcomes according to hypophosphatemia severity.

	Severe hypophosphatemia	moderate hypophosphatemia	Mild hypophosphatemia
N	21	153	150
Age (years)	52.48±19.76	55.52±17.93	56.47±18.41
Male sex (n, %)	12 (57.14%)	96 (62.75%)	95 (63.33%)
BMI (kg/m2)	25.44±5.38	27.96±6.09	28.08±7.10
APACHE II	20.73±5.16	20.95±7.19	19.66±6.67
SOFA24	8.56±2.90#	7.98±3.49*	6.92±2.88*#
ΔSOFA24-72	-1.33±2.35	-1.08±2.24	-1.05±2.81
Admission reason (n,%)			
Medical	14 (66.66%)	85 (55.56%)	79 (52.67%)
Surgical	1 (4.76%)	23 (15.03%)	33 (22%)
Trauma	6 (28.57%)	38 (24.84%)	33 (22%)
Obstetrics	-	1 (0.65%)	-
Transplantation	-	6 (3.92)	5 (3.33%)
Length of stay (days)	10.13±8.77	10.19±8.91	10.28±10.16
Length of ventilation (days)	9.38±8.63	8.94±8.51	8.78±9.55
Prolong ventilation (n, %)	9 (42.86%)	71 (46.41%)	62 (41.33%)
average daily energy deficit (Kcal/day)	-890.31±500.78	-985.40±576.51	-943.61±558.39
Death (n, %)	4 (19.05%)	20 (13.07%)	11 (7.33%)
Vent7Mort (n, %)	11 (52.38%)	84 (54.90%)	81 (54%)

p value is based on t-test or Mann-Whitney (as appropriate) for numerical variables and chi-square test for categorial variables. p value>0.05 for all comparisons but for those which are marked *#.

# p<0.04

* p<0.03

Table S2. Multivariate analysis for trauma patients only.

S2a. Mortality

Effect	Odd's Ratio	95% Confidence Limits	
Hypophosphatemia	1.000	0.058	17.241
Age	1.000	0.918	1.089
Female sex	1.000	0.050	20.113
BMI	1.000	0.829	1.206
APACHEE-II	1.000	0.771	1.298
SOFA24	1.000	0.580	1.724
ΔSOFA	1.000	0.615	1.627
Average daily energy deficit	1.000	0.997	1.003

S2b. Vent7Mortality

Effect	Odd's Ratio	95% Confidence Limits	
Hypophosphatemia	1.113	0.102	12.098
Age	0.996	0.930	1.067
Female sex	0.585	0.065	5.307
BMI	1.060	0.909	1.235
APACHEE-II	1.020	0.837	1.242
SOFA24	1.018	0.670	1.546
ΔSOFA	1.124	0.745	1.695
Average daily energy deficit	1.003	1.001	1.006
